# The Most Demanding Scenarios of Play in Basketball Competition From Elite Under-18 Teams

**DOI:** 10.3389/fpsyg.2020.00552

**Published:** 2020-04-21

**Authors:** Jairo Vázquez-Guerrero, Francisco Ayala, Franc Garcia, Jaime Sampaio

**Affiliations:** ^1^Sports Performance Area, FC Barcelona, Barcelona, Spain; ^2^Department of Sport Sciences, Sports Research Centre, Miguel Hernandez University of Elche, Elche, Spain; ^3^Research Center in Sports Sciences, Health Sciences and Human Development, CIDESD, CreativeLab Research Community, Vila Real, Portugal

**Keywords:** worst-case scenario, movement demands, game analysis, inertial movement sensors, team sport

## Abstract

The main purpose of this study was to describe the most demanding scenarios of match play in basketball through a number of physical demand measures (high-intensity accelerations and decelerations, relative distance covered, and relative distance covered in established speed zones) for four different rolling average time epochs (30, 60, 180, and 300 s) during an official international tournament. A secondary purpose was to identify whether there were significant differences in physical demand measures among playing positions (centers, guards, and forwards) and levels (two best classified teams in the tournament and remaining teams), match scoring (winning, losing, and drawing), and playing periods (match quarter) at the moment of the most demanding scenarios. Data were collected from 94 male under 18 (U18) elite basketball players (age: 17.4 ± 0.7 years; stature: 199.0 ± 11.9 cm; body mass: 87.1 ± 13.1 kg) competing in a Euroleague Basketball Tournament. Measures were compared via a Bayesian inference analysis. The results revealed the presence of position-related differences [Bayesian factor (BF) > 10 (at least strong evidence) and standardized effect size (δ) > 0.6 (at least moderate)] so that centers covered a lower relative distance at speed zone 1 and had lower high-intensity accelerations and decelerations than guards. However, the Bayesian analysis did not demonstrate the existence of significant differences in any physical demand measure in relation to the playing level, match scoring, and playing periods at the moment of the most demanding scenarios. Therefore, this study provides coaches and strength and conditioning specialists with a most demanding scenario reference on physical demands that can be used as an upper limit threshold in the training and rehabilitation monitoring processes.

## Introduction

Basketball is an intermittent, court-based team sport that requires players to perform a substantive number of repeated high-intensity movements such as accelerations and decelerations, changes of direction, high-speed running, jumping, and landing (Ostojic et al., 2006; Scanlan et al., 2011). Therefore, a fundamental task for coaches and strength and conditioning specialists is the design, implementation, and monitoring of training programs that allow them to ensure that basketball players are prepared to deal with the high-intensity periods of match play. Accordingly, the study of players’ physical demands using variables like distance covered at different speeds (walking and jogging, high-speed running, sprinting) and the number of high-intensity actions executed (accelerations and decelerations) during match play has been one of the most common research topics in the last decades (Klusemann et al., 2013; Fox et al., 2018; Svilar et al., 2018; Vázquez-Guerrero et al., 2019).

State-of-the-art microtechnology allows monitoring players’ locomotor movements and fosters the development of novel approaches to study sports performance based on the identification of the most demanding passages or scenarios of match play (also called worst-case scenarios) using different rolling average time durations. In particular, a novel approach has been recently suggested to describe the most demanding scenarios of match play through the use of the “peak scores” of relevant (in terms of sport performance) physical demand measures after having examined second by second all their scores using rolling average time epochs (e.g., 0–3, 1–4, 2–5 min). Some studies have demonstrated that the use of the traditional sequential (also called fixed-length or discrete time epochs) approach to calculate physical demands based on average values (e.g., 0–3, 3–6, 6–9 min) underestimates the periods of maximum exigence (up to ∼25%) in intermittent sports, when compared to the novel rolling average time epoch approach, due not only to its inability to capture fluctuations in physical demands but also to issues of reduced sampling resolution associated with using pre-defined fixed-time epochs (Varley et al., 2012; Furlan et al., 2015). For example, whether the most demanding scenario of a particular physical demand measure occurred between 2 and 5 min during competitive match play, the use of the traditional segmental analysis that take averages from 0–3 and 3–6 min would miss the full peak period, and this consequently would lead to an underestimation of this physical demand measure (Whitehead et al., 2018). These observed differences in players’ physical demands between the sequential and rolling average time epoch approaches seem to increase as the time epoch length decreases (i.e., below 5 min), which may be due to the physiological, contextual, and technical–tactical demands of the sport (Ly et al., 2016; Wagenmakers et al., 2018). Furthermore, it has been also suggested that the higher the sample frequency is, the larger the inter-approach differences may be (Doncaster et al., 2019).

Therefore, this new rolling average time epoch approach, unlike the studies reporting the average physical demands of match play, may provide greater insight into the requirements on players during the most intense active phases of matches. In terms of practical applications, knowing the most demanding scenarios of match play in intermittent team sports may be especially relevant for the development of ecologically valid physical stimuli during training drills that ensure that players are appropriately prepared for the most demanding periods (in terms of physical demands) of match play (Gabbett et al., 2012; Gabbett, 2016; Tierney et al., 2017). Likewise, the characterization of the most demanding scenarios in physical demands of match play might also improve the rehabilitation programs due to the fact that restoring players’ specific fitness and locomotor performance in relation to match physical demands may be a primary return-to-play criterion from a sport-related injury (Buchheit and Mayer, 2018).

While some studies have determined the most demanding scenarios in physical demands during competition in intermittent team sports such as association football (Abbott et al., 2018; Delaney et al., 2018; Martín-García et al., 2018; Casamichana et al., 2019), rugby (Delaney et al., 2016; Cunningham et al., 2018), Gaelic football (Malone et al., 2017), and Australian football (Delaney et al., 2017) through different time average rolling durations, no studies are available that quantify physical demands during match play in basketball using this approach. A plethora of studies have examined the average (mainly per minute) and absolute physical demands of match play in basketball reporting that players usually cover 5–6 km at an average speed of 70–90 m min^–1^ and perform a total of 40–50 jumps (Stojanović et al., 2018). Furthermore, most of these studies have also identified that the physical demands experienced by players during basketball match play are influenced by the playing positions and levels, whereby guards and top players sustain greater workloads than forwards, centers, and lower-level players at the same positions. However, as stated before, the utility of this information to develop appropriate training programs to optimize physical preparedness for competition may be limited.

Therefore, the main purpose of this study was to describe the most demanding scenarios of match play in basketball through a number of physical demand measures for four different rolling average time epochs (30, 60, 180, and 300 s) during an official U18 international tournament. A secondary purpose was to identify whether there were differences in physical demands among playing positions (centers, guards, and forwards) and levels (two best classified teams in the tournament and remaining teams), match scoring (winning, losing, and drawing), and playing periods (match quarter) at the moment of the most demanding scenarios.

## Materials and Methods

### Participants

A total of 94 male (under 18) elite basketball players (age: 17.4 ± 0.7 years; stature: 199.0 ± 11.9 cm; body mass: 87.1 ± 13.1 kg) from eight teams and six countries competing in the 2017 edition of the Euroleague Basketball Next Generation Tournament participated in the current study. Players were grouped according to their playing position as centers (*n* = 17), guards (*n* = 35), and forward (*n* = 42) (Abdelkrim et al., 2010a,b). Playing positions were first determined by the information available on the Euroleague website and further refined by a qualified basketball coach (i.e., level 3 certificate in coaching basketball from the Spanish Basketball Federation with more than 5 years of experience coaching teams in top national leagues). Before any participation, experimental procedures and potential risks were fully explained to both players and coaches in verbal and written forms, and written informed consent was obtained from them. The experimental procedures used in this study were in accordance with the Declaration of Fortaleza and were approved by the local Ethics and Scientific Committee.

### Procedures

A descriptive study design was used to address the purposes of this study. All 13 matches from the tournament were monitored over the 4-day schedule. The matches were played on the same court in similar environmental conditions at different times of the day according to the official schedule and using International basketball federation official rules. Matches started with a 15 min warm-up. During each match play, all the players were continuously monitored, but the physical demands were quantified only when players were competing on court (e.g., when a player was a substitute or when there was a rest time between quarters, this was not included). All players were not required to follow any previous dietary recommendation or restriction, but they were able to replace water loss by drinking *ad libitum* during the game recovery periods.

Players’ data were included for analysis provided they did not suffer injury during the match, played in the same position throughout the match, and played at least 5 min of live time in each match (Sampaio et al., 2006, 2010).

Players’ movements were measured using a portable local positioning system (LPS) (WIMU PRO^®^, Realtrack Systems SL, Almería, Spain) during matches. Devices (81 mm × 45 mm × 15 mm, 70 g) were fitted to the upper back of each player using an adjustable harness (Rasán, Valencia, Spain). The WIMU PRO units integrate different sensors registering at different sample frequencies. Sampling frequency for a three-axis accelerometer, gyroscope, and magnetometer was 100 Hz and 120 kPa for the barometer. The system has six ultra-wide-band antennas, four placed 3 m outside the corners of the court and two placed 3 m outside half-court; the sampling frequency for positioning data was 20 Hz. The system operates using triangulations between the antennas and the units; the six antennas send a signal to the units every 50 ms. Then, the device calculates the time required to receive the signal and derives the unit position (coordinates x and y), using one of the antennas as a reference.

WIMU PRO^®^ software was used for the computation of rolling averages over each physical demand measure of interest using four different time epochs (30, 60, 180, and 300 s), and the maximum value for each time epoch was recorded. For example, for a 60 s rolling average with a sampling of 20 Hz, the software identified 1,200 consecutive data points (i.e., 18 samples/s for 60 s). For a 120 s rolling average, 2,400 samples were used, and so on. Thus, for the 60 s rolling epoch, algorithm values were calculated using the current and the 1,180 preceding samples. A similar procedure was followed for the four different time epochs selected.

In each time epoch, the peak values of the physical demand measures selected were recorded independently, so that it is very likely that they came from different data points. The rationale behind the selection of the 30, 60, and 180 s time epochs was based on the findings shown by previous studies that report that: a) approximately 90% of the live-time actions in basketball match play have a duration lower than 80 s (of which around 17 and 26% exhibited a duration near 30 and 60 s, respectively) and b) actions with durations longer than 180 s are very unlikely (Conte et al., 2016; Zhang et al., 2019). Time frames shorter than 30 s were considered too short from a task design perspective and consequently were discarded.

Although the 300 s time epoch does not represent the most common durations of the actions that characterize the game of basketball, it was finally selected because after having spoken with several coaches and strength and conditioning specialists, most of them agreed that this duration may represent the time that several players are on court during basketball match play before being substituted or a time-out is provided. Therefore, describing the most demanding scenarios in physical demands during competition using this wide time epoch also might be useful to know the peak physical demands that a player usually has to address before a stoppage time phase higher than 40 s is given.

Therefore, for each match play, maximum values using 10 physical demand measures were calculated for each time epoch. In particular, the following physical demand variables were measured and reported: (a) relative distance (total distance/playing duration); (b) relative distance in established speed zones [zone 1: stationary/walking (<6.0 km h^–1^), zone 2: jogging (6.0–12.0 km h^–1^), zone 3: running (12.1–18.0 km h^–1^), zone 4: high-intensity running (18.1–24.0 km h^–1^), and zone 5: sprinting (>24.0 km h^–1^)]; c) high-intensity accelerations (>2 m s^–2^) and decelerations (<-2 m s^–2^); and f) distance covered at high-intensity accelerations (>2 m s^–2^) and decelerations (<-2 m s^–2^). The speed and movement zones selected were similar to those used in other basketball studies (McInnes et al., 1995; Puente et al., 2017).

The LPS showed acceptable accuracy for measures of speed and mean acceleration and deceleration for intermittent activities (Stevens et al., 2014). The WIMU PRO^®^ system showed better accuracy (bias: 0.57–5.85%), test–retest reliability (%TEM: 1.19), and inter-unit reliability (bias: 0.18) in determining distance covered compared to GPS technology (bias: 0.69–6.05%; %TEM: 1.47; bias: 0.25) overall when both devices were worn by the same athlete (Bastida-Castillo et al., 2018). More recently, the WIMU PRO^®^ system showed a mean absolute error of 5.2 ± 3.1 cm for the x-position and 5.8 ± 2.3 cm for the y-position. This represents percentage of differences of 0.97 ± 1% for the x-coordinate and 0.94 ± 1.14% for the y-coordinate (Bastida-Castillo et al., 2019). The inter-unit reliability showed a large ICC for the x-coordinate (0.65) and a very large ICC for the y-coordinate (0.88), and a good%TEM (2%) was reported for the error agreement between the two devices assessed.

To compare different playing levels, the database was divided into (a) top teams, defined as the two teams that reached the final of the tournament, and the (b) remaining six teams.

### Statistical Analysis

Statistical analyses were performed using JASP (Amsterdam, Netherland) software version 0.10. Data are presented as mean and 95% credible intervals. In order to analyze the possible effects of the fixed factors [time epoch (30, 60, 180, and 300 s), playing positions (center, guard, and forward) and levels (two best classified teams and the remaining six teams), playing period (first quarter, second quarter, third quarter, and fourth quarter), and match scoring (winning, losing, and drawing) at the moment of the most demanding scenarios] on the dependent variables previously described [high-intensity accelerations (number and distance covered) and decelerations (number and distance covered), relative distance covered, and relative distance covered in established speed zones], separate ANOVAs were conducted using a Bayesian statistical approach. Individual “player code” was treated as a random factor for all analysis.

The Bayesian methodology [based on the quantification of the relative degree of evidence for supporting two rival hypotheses, null hypothesis (H_0_) vs. alternative hypothesis (H_1_), by means of the Bayesian factor (BF_10_) (Linke et al., 2018; Doncaster et al., 2019)] has been recently suggested as an alternative to the traditional frequentist statistics (based on confidence intervals and p values) for hypothesis testing due to (among others) the following benefits: the BF_10_ quantifies evidence that the data provide for H_0_ vs. H_1_, the BF_10_ can quantify evidence in favor of H_0_, and the BF_10_ is not “violently biased” against H_0_ (Ly et al., 2016; Wagenmakers et al., 2018).

The BF_10_ was interpreted using the evidence categories suggested by Lee and Wagenmakers (2013): $<\frac{1}{100}$ = extreme evidence for H_0_, from$\frac{1}{100}$ to$<\frac{1}{30}$ = very strong evidence for H_0_, from $\frac{1}{30}$ to $<\frac{1}{10}$ = strong evidence for H_0_, from $\frac{1}{10}$ to $<\frac{1}{3}$ = moderate evidence for H_0_, from $\frac{1}{3}$ to <1 anecdotal evidence for H_0_, from 1 to 3 = anecdotal evidence for H_1_, from >3 to 10 = moderate evidence for H_1_, from >10 to 30 = strong evidence for H_1_, from >30 to 100 = very strong evidence for H_1_, and >100 extreme evidence for H_1_.

In order to provide a high probability of obtaining compelling evidence, only those ANOVAs that showed at least strong (10 times higher) evidence for supporting H_1_ (BF_10_ > 10) and a percent error <0.001 (which indicates great stability of the numerical algorithm that was used to obtain the result) were considered robust enough to identify true differences between models (null model vs. factor-specific model), and posterior *post hoc* analyses were then carried out. Paired comparisons were based on either the Bayesian independent samples t-test (for normally distributed variables) with a Cauchy prior (0, *r* = $\frac{1}{\sqrt{2}}$) or the Bayesian Mann–Whitney U test (for non-normally distributed variables). The distribution of raw data sets was checked through the Shapiro–Wilk Expanded test. The posterior odds were corrected for multiple testing by fixing to 0.5 the prior probability that the null hypothesis holds across all comparisons (Westfall et al., 1997). Again, a BF > 10 was needed to consider a difference in any paired comparison as significant.

The median and the 95% central credible interval of the posterior distribution of the standardized effect size (δ) (i.e., the population version of Cohen’s *d*) were also calculated for each of the paired comparisons carried out. Magnitudes of the posterior distribution of the standardized effect size were classified as: trivial (<0.2), small (>0.2–0.6), moderate (>0.6–1.2), large (>1.2–2.0), and very large (>2.0–4.0) (Batterham and Hopkins, 2006).

From a training prescription standpoint, small changes in the physical demand measures selected in the current study are unlikely to influence a coach’s prescription of training drills. Therefore, this study established the following requirements that needed to be fulfilled in order to infer that a difference noted between paired comparisons across the different fixed factors in the physical demand measures recorded was substantial or relevant from the perspective of ensuring a proper design of training tasks: (a) BF_10_ > 10 (at least strong evidence for supporting H_1_) and (b) δ > 0.6 (at least moderate).

## Results

A total of 29,867 observations (i.e., peak scores) from 10 physical demand measures, 13 matches, 94 players, and four different time epochs were collected.

Table 1 displays the descriptive statistics of the dependent variables recorded for each time epoch. For the Bayesian ANOVA conducted with the time epoch as a fixed factor, the results showed extreme evidence (BF > 100 and percentage error <0.001) that supports the existence of a main effect for time epoch for all movement demand variables (with the exception of the relative *distance covered at speed zone 4* and relative *distance covered at speed zone 5* variables) (Table 1). The subsequent *post hoc* analysis revealed substantial differences (BF > 10 and δ > 0.6) in values for each dependent variable across all time epochs [with the exception of the *distance covered at speed zone 3 (running)*].

**TABLE 1:** The most demanding scenario of basketball match play for four different time epochs (mean and 95% credible intervals).

Variable	Time epochs							
	30 s		60 s		180 s		300 s	
Accelerations*								
Distance covered (m)	26.5	(25.9–26.9)**^b,c,d^**	35.9	(35.2–36.6)**^a,c,d^**	65.7	(64.3–67)**^a,b,d^**	87.7	85.8–89.5**^a,b,c^**
Number	4.9	(4.8–5)**^b,c,d^**	6.9	(6.8–7)**^a,c,d^**	13.3	(13.1–13.6)**^a,b,d^**	18	(17.7–18.3)**^a,b,c^**
Decelerations*								
Distance covered (m)	23.3	(22.8–23.8)**^b,c,d^**	32.7	(31.8–33.5)**^a,c,d^**	53.7	(52.5–54.9)**^a,b,d^**	71.2	(69.5–72.8)**^a,b,c^**
Number	4.6	(4.5–4.7)**^b,c,d^**	6.4	(6.3–6.5)**^a,c,d^**	11.8	(11.6–12.1)**^a,b,d^**	16	(15.7–16.3)**^a,b,c^**
Relative distance covered*	71.8	(71–75.5)**^b,c,d^**	120.4	(119.2–121.6)**^a,c,d^**	282.6	(279.6–285.6)**^a,b,d^**	428.6	(422.4–434.8)**^a,b,c^**
**Relative distance covered at different speed zones (m)**								
Zone 1 (stationary/walking)	62.1	(61.3–62.9)**^b,c,d^**	94.9	(93.5–96.2)**^a,c,d^**	193.8	(190.7–196.8)**^a,b,d^**	273.6	(269.5–278.1)**^a,b,c^**
Zone 2 (jogging)	38.2	(37.4–38.9)**^b,c,d^**	48.7	(47.5–49.7)**^a,c,d^**	88.9	(86.9–90.9)**^a,b,d^**	119.8	(116.9–122.6)**^a,b,c^**
Zone 3 (running)	13.6	(13.1–14)^b,^**^c,d^**	15	(14.5–15.4)^a,^**^c,d^**	20.1	(19.4–20.9)**^a,b^**^,d^	24.1	(23.1–25.1)**^a,b^**^,c^
Zone 4 (high-intensity running)	8.2	(7.8–8.5)	8.4	(8–8.8)	9.5	(9–9.9)	10.3	(9.7–10.8)
Zone 5 (sprinting)	5.7	(5.2–6.3)	5.8	(5.2–6.3)	6.1	(5.5–6.7)	6.2	(5.6–6.9)

*^∗^The Bayesian ANOVA reported that there was at least a strong evidence (Bayes factor [BF_10_] > 10 and percent error <0.001) to support the alternative hypothesis (H_1_). *Post hoc* analysis: super-indices indicate that there was at least a strong evidence to support the presence of differences (BF_10_ > 10) against ^*a*^30 s time epoch, ^*b*^60 s time epoch, ^*c*^180 s time epoch and ^*d*^300 s time epoch. In bold are emphasized those sub-indices whose magnitude of the differences observed were at least moderate (δ > 0.6). m: meters; s: seconds; km: kilometer; h: hour.*

In Tables 2 and 3 are presented the means and 95% credible intervals of the dependent variables in the four selected time epochs and separately for each player specific position and playing level, respectively. Likewise, in these tables are also shown their respective main ANOVA results. When playing position was included as a fixed factor in the Bayesian ANOVA, there was at least moderate evidence that supported the presence of significant positional differences in the following variables: accelerations (distance covered and number), decelerations (distance covered and number), relative distance at speed zone 1 (stationary/walking), and relative distance at speed zone 2 (jogging). Specifically, the *post hoc* analysis showed that centers had lower scores in the aforementioned variables than forward and guards. However, the magnitudes of these differences were only substantial (BF > 10 and δ > 0.6) for the paired comparison between centers and guards in the acceleration [distance (from -9.7 to -23.3 m) and number (from -2.4 to -4.8)], deceleration [distance (from -14.6 to -26.5 m) and number (from -3.9 to -6.2)], and relative distance at speed zone 1 (from -33.1 to -65.7 m) variables registered using a 300 s time epoch. Likewise, substantial differences between centers and guards were also found in the numbers of high-intensity decelerations for the 60 s (from -1.2 to -2.1) and 180 s (from -2.8 to -4.4) time epochs and in the relative distance at speed zone 1 for the 180 s time epoch (from -18.3 to -40.5 m). For its part, the Bayesian analysis did not demonstrate the existence of significant differences in any dependent variable in relation to the playing level (BF < 10).

**TABLE 2:** The most demanding scenario of basketball match play for three different playing positions (mean and 95% credible intervals).

Time epoch (s)	Playing positions					
	Center		Forward		Guard	
Accelerations [distance covered (m)]*						
30	23.5	(22.3–24.7)^b,c^	26.8	(26–27.6)^a^	27.1	(26.4–27.8)^a^
60	32.1	(30.4–33.8)^b,c^	36.1	(35.1–37.2)^a^	37	(35.9–38.1)^a^
180	57.9	(54.3–61.4)^b,c^	65.3	(63.2–67.4)^a^	68.5	(66.5–70.5)^a^
300	75.8	(70.8–80.8)^b,c^	86.7	(83.9–89.5)^a^	92.3	(89.6–94.9)^a^
Accelerations (number)*						
30	4.4	(4.2–4.6)^b,c^	4.8	(4.7–4.9)^a^	5.1	(5–5.3)^a^
60	6.2	(5.9–6.5)^c^	6.7	(6.6–6.9)^c^	7.3	(7.2–7.5)^a,b^
180	11.7	(11.1–12.4)^b,c^	12.9	(12.6–13.3)^a,c^	14.2	(13.9–14.6)^a,b^
300	15.7	(14.8–16.6)^b,c^	17.4	(16.9–17.9)^a,c^	19.3	(18.8–19.8)^a,b^
Decelerations [distance covered (m)]*						
30	20.4	(19.3–21.6)^b,c^	23.2	(22.5–23.9)^a^	24.4	(23.7–25.1)^a^
60	29.1	(26.8–31.3)^c^	32.3	(31–33.6)	34.3	(33.1–35.6)^a^
180	44.1	(41.2–47)^b,c^	52.8	(50.9–54.7)^a,c^	57.6	(55.9–59.4)^a,b^
300	56.4	(52.4–60.4)^b,c^	69.8	(67.3–72.4)^a,c^	76.9	(74.7–79.2)^a,b^
Decelerations (number)*						
30	3.9	(3.7–4.2)^b,c^	4.5	(4.4–4.6)^a,c^	4.9	(4.8–5.1)^a,b^
60	5.3	(5.1–5.6)^b,c^	6.1	(5.9–6.3)^a,c^	6.9	(6.8–7.2)^a,b^
180	9.4	(8.9–9.9)^b,c^	11.4	(11.1–11.7)^a,c^	13.1	(12.7–13.4)^a,b^
300	12.7	(11.9–13.5)^b,c^	15.2	(14.8–15.7)^a,c^	17.7	(17.2–18.2)^a,b^
Relative distance covered (m)*						
30	67.2	(65.4–69.1)^b,c^	71.4	(70.2–72.6)^a^	73.5	(72.3–74.6)^a^
60	113	(110.4–115.7)^b,c^	120	(117.9–121.6)^a^	123.4	(121.7–125.3)^a^
180	261.5	(253.6–269.4)^b,c^	282.6	(278–287.2)^a^	289.5	(285.1–293.9)^a^
300	394.7	(375.6–413.7)^b,c^	429	(419.2–438.8)^a^	438.9	(430.7–447.2)^a^
Relative distance covered at the speed zone 1 (stationary / walking) (m)*						
30	56.1	(54.2–58.1)^b,c^	62.4	(61.2–63.6)^a^	63.9	(62.6–65.2)^a^
60	84.9	(81.7–88)^b,c^	94.9	(92.9–96.9)^a^	98.2	(96.1–100.2)^a^
180	171.9	(164.5–179.4)^b,c^	193.3	(188.5–197.9)^a^	201.3	(196.9–205.8)^a^
300	237.2	(226.2–248.2)^b,c^	272.2	(265.2–279.1)^a^	286.6	(280.2–293.1)^a^
Relative distance covered at the speed zone 2 (jogging) (m)*						
30	34.3	(32.4–36.2)^b,c^	38.9	(37.7–40.1)^a^	38.8	(37.6–39.9)^a^
60	43.1	(40.4–45.7)^b,c^	50.4	(48.7–52)^a^	48.8	(47.1–50.6)^a^
180	77.9	(73.3–82.7)^b,c^	90.3	(87–93.6)^a^	91.1	(88–94.2)^a^
300	103.7	(96.9–110.4)^b,c^	121.1	(116.5–125.6)^a^	123.7	(119.6–127.8)^a^
**Relative distance covered at the speed zone 3 (running) (m)**						
30	12.7	(11.6–13.7)	13.5	(12.8–14.1)	13.9	(13.3–14.5)
60	13.8	(12.6 – 15)	14.9	(14.1–15.7)	15.4	(14.7–16.1)
180	17.5	(15.8–19.3)	20.2	(18.9–21.4)	20.8	(19.7–21.9)
300	21.1	(18.6–23.5)	23.9	(22.3–25.5)	25.1	(23.7–26.5)
**Relative distance covered at the speed zone 4 (high-intensity running) (m)**						
30	7.6	(6.6–8.6)	8.2	(7.7–8.8)	8.3	(7.8–8.8)
60	7.7	(6.7–8.8)	8.5	(7.9–9.1)	8.5	(7.9–9.1)
180	8.3	(7.1–9.6)	9.6	(8.9–10.4)	9.5	(8.8–10.3)
300	9.2	(7.6–10.8)	10.3	(9.4–11.2)	10.5	(9.6–11.3)
**Relative distance covered at the speed zone 5 (sprinting) (m)**						
30	6.1	(3–9.3)	5.5	(4.9–6.2)	5.7	(4.9–6.4)
60	6.2	(3–9.3)	5.5	(4.9–6.2)	5.9	(5–6.7)
180	6.6	(3.3–9.9)	5.9	(5.2–6.5)	6.2	(5.2–7.2)
300	6.9	(3.7–10.3)	5.9	(5.2–6.6)	6.4	(5.4–7.4)

*The Bayesian ANOVA reported that there was at least a strong evidence [Bayes factor (BF_10_) > 10] to support the alternative hypothesis (H_1_) and percent error <0.001. *Post hoc* analysis: super-indices indicate that there was at least a strong evidence to support the presence of differences (BF_10_ > 10) against ^*a*^center, ^*b*^forward, and ^*c*^guard. In bold are emphasized those sub-indices whose magnitude of the differences observed were at least moderate (δ > 0.6). m: meters; s: seconds; km: kilometer; h: hour.

**TABLE 3:** The most demanding scenario of basketball match play for two different playing levels (mean and 95% credible intervals).

Time epoch (s)	Playing levelg			
	Two best teams		Remaining teams	
**Accelerations [distance covered (m)]**				
30	26.4	(25.5–27.3)	26.5	(25.9–27.1)
60	35.9	(34.7–37.1)	35.9	(35.1–36.8)
180	65.6	(63.2–68.1)	65.7	(64–67.3)
300	89.8	(86.5–93.1)	86.8	(84.5–89)
**Accelerations (number)**				
30	5	(4.9–5.2)	4.9	(4.8–4.9)
60	7.2	(6.9–7.4)	6.8	(6.7–6.9)
180	13.9	(13.5–14.4)	13.1	(12.8–13.3)
300	18.9	(18.2–19.5)	17.7	(17.3–18.1)
**Decelerations [distance covered (m)]**				
30	22.8	(21.9–23.6)	23.6	(23–24.2)
60	33.8	(32.2–35.5)	32.2	(31.3–33.2)
180	52.6	(50.6–54.6)	54.2	(52.6–55.7)
300	71.2	(68.4–74.1)	71.1	(69.1–73.2)
**Decelerations (number)**				
30	4.7	(4.5–4.8)	4.6	(4.5–4.7)
60	6.5	(6.2–6.7)	6.4	(6.2–6.5)
180	12.2	(11.8–12.6)	11.7	(11.4–11.9)
300	16.6	(15.9–17.2)	15.7	(15.3–16.1)
**Relative distance covered (m)**				
30	72.4	(71.1–73.7)	71.5	(70.6–72.5)
60	122.2	(120.1–124.2)	119.7	(118.2–121.1)
180	285.6	(280.2–290.9)	281.4	(277.8–285.1)
300	426.2	(418.1–434.2)	429.7	(421.6–437.8)
**Relative distance covered at the speed zone 1 (stationary/walking) (m)**				
30	61.9	(60.4–63.6)	62.2	(61.3–63.2)
60	95.8	(93.4–98.2)	94.5	(92.9–96.1)
180	195.6	(190.1–201.1)	193	(189.4–196.7)
300	276.4	(268.6–284.2)	272.4	(266.9–277.9)
**Relative distance covered at the speed zone 2 (jogging) (m)**				
30	37.6	(36.2–38.9)	38.4	(37.5–39.4)
60	45.1	(42.9–47.4)	50.1	(48.8–51.4)
180	89.1	(85.5–92.8)	88.8	(86.3–91.3)
300	120.8	(116.1–125.5)	119.4	(115.9–122.9)
**Relative distance covered at the speed zone 3 (running) (m)**				
30	13.97	(13.3–14.7)	13.4	(12.9–13.9)
60	15.44	(14.6–16.3)	14.7	(14.2–15.3)
180	21.17	(19.8–22.5)	19.7	(18.7–20.6)
300	25.91	(24.1–27.7)	23.3	(22.1–24.4)
**Relative distance covered at the speed zone 4 (high-intensity running) (m)**				
30	8.22	(7.5–8.9)	8.2	(7.8–8.6)
60	8.42	(7.7–9.2)	8.4	(7.9–8.9)
180	9.54	(8.6–10.5)	9.4	(8.9–10)
300	10.86	(9.7–11.9)	10	(9.3–10.7)
**Relative distance covered at the speed zone 5 (sprinting) (m)**				
30	6.66	(5.5–7.8)	5.2	(4.8–5.7)
60	6.76	(5.6–7.9)	5.3	(4.8–5.8)
180	7.28	(5.9–8.7)	5.5	(4.9–6.1)
300	7.55	(6.1–9)	5.6	(5–6.3)

Supplementary Appendices 1 and 2 show the values of the dependent variables in the four selected time epochs and separately for each playing period and match scoring at the moment of the most demanding scenarios. Despite the fact that the Bayesian analysis reported (with at least a strong degree of evidence) that in the first quarter, the scores of the high-intensity accelerations (distance covered and number) and decelerations (distance covered and number) and relative distance covered at speed zone 1 measured for the 300 s time epoch were higher than their counterparts showed in the last quarter (and in certain occasions, they were also lower than in the third quarter), the magnitude of these differences (δ < 0.6) was not large enough to overcome the cutoff score established to fulfill the second requirement needed to consider any change as substantial. Similarly, there was not enough evidence to support any relevant effect elicited by the factor of match scoring at the moment of the most demanding scenarios on the physical demand measures analyzed (Supplementary Appendix 2).

## Discussion

The current study provides novel results that may help coaches and strength and conditioning specialists to better understand the most demanding scenarios of basketball games and, thus, improve evidence-based approaches when designing effective training and rehabilitation interventions. In particular, this study has described the most demanding scenarios of competitive basketball match play from under-18 teams through 10 physical demand measures and using four different rolling average time epochs: 30, 60, 180, and 300 s. Thus, and for example, the results showed that for the 60 s time epoch, the players performed 6.9 and 6.4 high-intensity accelerations and decelerations for a total covered distance of 120.4 m. Therefore, coaches and sports science specialists, when designing training drills with a duration of 60 s, should monitor that players achieve these just-mentioned peak physical demands in order to ensure proper preparation for what they will face during competitive match play. It should be also highlighted that as there were substantial differences (BF > 10 and δ > 0.6) in values for most of the dependent variables across all time epochs, coaches and strength and conditioning specialists are advised to align the epoch length to the duration of the specific training drill that is being monitored or prescribed. For example, the number of high-intensity accelerations from a 60 s time epoch should not be extrapolated and utilized to assess and/or prescribe training drills that are longer (e.g., 90 s) or shorter (e.g., 45 s) in duration.

This activity profile described in the current study seems much more demanding than those found in previous studies using average measures instead of rolling average time epochs (Abdelkrim et al., 2007; Vázquez-Guerrero et al., 2019). In particular, Vázquez-Guerrero et al. (2019) showed that basketball players averaged 1.8 and 1.5 high-intensity accelerations and decelerations and covered a relative distance of 72.6 m min^–1^. A similar trend has been observed in previous studies conducted in other sports, such as associated football (Abbott et al., 2018; Delaney et al., 2018; Martín-García et al., 2018; Casamichana et al., 2019), rugby (Delaney et al., 2016; Cunningham et al., 2018), Gaelic football (Malone et al., 2017), and Australian football (Delaney et al., 2017).

Another important finding reports that during the most demanding scenarios, the physical demands of match play are position-dependent (with the exception of the relative distances covered at speed zones 3, 4, and 5), the magnitude of these differences being larger for the longer time epochs. Consequently, it may be suggested that reducing the time epoch homogenizes the physical demands imposed on players. Thus, the centers performed a lower amount of high-intensity accelerations and decelerations and covered less distance than the forward, and much less than the guards. However, only the differences found between the centers and the guards for the high-intensity accelerations (number and distance covered) and decelerations (number) and relative distance covered at speed zone 1 measured at the 180 and 300 s time epochs may be considered relevant from a training prescription standpoint (BF > 10 and δ > 0.6). These results may partly be explained by the inherent tactical and technical requirements of the playing positions, players’ individual physical demands, and the team play model (Abdelkrim et al., 2007). In fact, guards performed the highest number of high-intensity accelerations and decelerations presumably because these positions require a wide diversity of tactical movements such as hand-off, picks, and screen actions. Conversely, the lower amount of high-intensity accelerations and decelerations in centers could reflect the specificity of positional roles’ actions, where they are required to occupy smaller spaces located nearer the basket (Sampaio et al., 2006). Similar playing position–related differences in physical demands of competition in basketball were found in previous studies using average scores (Ferioli et al., 2018; Stojanović et al., 2018; Svilar et al., 2018). Therefore, and unlike short-duration tasks, when coaches and sports science specialists want to design conditioning drills with a duration of 180 and 300 s and that replicate match physical demands, the positional differences should be taken into consideration so that the guards are subjected to higher physical demands (in terms of accelerations and decelerations and distance covered) than the forwards (e.g., two or three accelerations and decelerations more) but mainly compared to the centers (e.g., at least five accelerations and decelerations more). The activities that most likely can achieve this effect seem to be the small-sided game situations, because it’s possible in these tasks to preserve match informational characteristics and have the players attending to the overall needs imposed by their roles. In these situations, it should be noted that coaches might benefit from adjusting interventions to instruct the players according to the time epochs. For example, if targeting a small-sided game situation with the most demanding scenario at a time epoch of 180 s, any game stoppage should be minimized or even completely avoided.

Regarding the playing level (i.e., tournament outcome), the results of this study did not show substantial differences between the two teams that played the final and the rest of the teams in any physical demand measure recorded during the most demanding scenarios of match play. This circumstance suggests that increased higher-intensity activity is not a decisive factor for winning. At this level of play, all teams reaching this stage of the tournament have already shown quality of play; therefore, it might be likely that a team unable to keep intense game paces would not reach the tournament qualification.

Similarly, this study did not find substantial differences in the physical demand measures selected according to the playing period (match quarter) at the moments of the most demanding scenarios. It might suggest that players may not have experienced enough fatigue that impaired physical performance during the game, as the rules allow for unlimited substitutions. Contrarily, previous research showed a significant reduction in averaged relative distance and high-intensity accelerations and decelerations especially between the first and last quarters for players in all playing positions (Abdelkrim et al., 2007, 2010a; Vázquez-Guerrero et al., 2019; García et al., 2020). These contradictory results could be attributed to a methodological issue. In this sense, it has been reported that the fourth quarter of basketball match play [similar to the second half of football match play (Linke et al., 2018)] usually presents a longer total playing time [the sum of ball-in-play time (also called effective play time) and time dedicated to all game stoppages] than the first quarter (approximately 10 min more), mainly due to the higher number of substitutions, fouls, time-outs, and other actions that require interrupting the game (Scanlan et al., 2019). These game interruptions may have caused the just-mentioned reductions in averaged relative distance and high-intensity accelerations and decelerations between the first and fourth quarters observed in previous studies reporting the average physical demands of basketball match play. When a rolling average time epoch approach is used to describe the peak physical demands of play in intermittent team sports competitions (including basketball), game interruptions is not a factor that can bias the results.

Finally, when comparing the most demanding scenarios for the physical demands and the scoring for each quarter, no strong statistical evidence appeared except for the distance covered at >12 km/h at the 300 s time epoch. It might suggest that physical demands in basketball are not dependent on the score during periods less than 5 min. Moreover, it could also indicate that both teams adjust the physical demands according to the opponent’s team play, not depending on the score.

Further expansions of this study can be done by including individual information about the players, such as physical fitness measures that help to understand the players’ maximal possibilities. The analysis of this study was done under a single tournament with congested fixtures; thus, the findings shown may not be extrapolated to describe the most demanding scenarios in preseason, season, and playoff moments. For technical reasons, the most demanding scenarios were calculated for each player without considering the rest of his teammates and opponents at the same moment, and consequently, we were not able to analyze the effect of having (or not) ball possession (i.e., offense vs. defense match situation) and temporal changes in the team’s and opposition’s tactics and playing system on the most demanding scenarios of play in basketball competition. Future studies are warranted to address these issues.

## Conclusion

The current study provided results from a high-level cohort of young basketballers, describing the most demanding scenarios of match play using time epochs of 30, 60, 180, and 300 s. The main practical application for coaches and strength and conditioning professionals is that most demanding scenarios can be used as an upper limit threshold in the training monitoring process. In fact, preparation to play high-level basketball requires the ability to perceive and act over the environment at extremely high game paces. Likewise, having these upper limits well defined is also a step forward in improving evidence-based approaches in the process of returning to play after injury.

## Data Availability Statement

The datasets generated for this study are available on request to the corresponding author.

## Ethics Statement

The studies involving human participants were reviewed and approved by the Research Center in Sports Sciences, Health and Human Development (UID/DTP/04045/2013). Written informed consent to participate in this study was provided by the participants’ legal guardian/next of kin.

## Author Contributions

JV-G participated in the design of the study, contributed to data collection and interpretation of results. FA contributed to data reduction/analysis and interpretation of results. FG participated in the design of the study and contributed to data collection. JS contributed to data reduction/analysis. All authors contributed to the manuscript writing, read and approved the final version of the manuscript, and agreed with the order of presentation of the authors.

## Conflict of Interest

JV-G and FG were employed by FC Barcelona. The remaining authors declare that the research was conducted in the absence of any commercial or financial relationships that could be construed as a potential conflict of interest.
