# Supplementary material for: The Most Demanding Scenarios of Play in Basketball Competition From Elite Under-18 Teams
Source: Front Psychol. 2020 Apr 21;11:552. doi: 10.3389/fpsyg.2020.00552 (PMC7187750; doi:10.3389/fpsyg.2020.00552)
Supplement: Supplementary file 1 [file Data_Sheet_1.docx]

| **Appendix 1: The most demanding scenario of a basketball match play that were recorded in each playing period (mean and 95% credible intervals)** | | | | | | | | |
| --- | --- | --- | --- | --- | --- | --- | --- | --- |
| **Time epoch** | **10-minute quarters** | | | | | | | |
|  | **Quarter 1** | | **Quarter 2** | | **Quarter 3** | | **Quarter 4** | |
|  | Accelerations (distance covered [m]) | | | | | | | |
| 30 s | 27.5 | (26.5 - 28.4) | 27.2 | (26.2 - 28.2) | 25.9 | (25 - 26.9) | 25.1 | (24.1 - 26) |
| 60 s | 37.6 | (36.4 - 38.9) | 36.2 | (34.8 - 37.5) | 35.3 | (33.9 - 36.8) | 34.6 | (33.1 - 36.1) |
| 180 s* | 69.9 | (67.3 - 72.6) ^d^ | 65.7 | (63 - 68.4) | 65.1 | (62.3 - 67.9) | 61.6 | (58.8 - 64.3) ^a^ |
| 300 s* | 96.4 | (92.6 - 100.2) ^b,c,d^ | 86.7 | (83.3 - 90.2) ^a^ | 85.8 | (82.3 - 89.2) ^a^ | 81.5 | (77.7 - 85.2) ^a^ |
|  | Accelerations (number) | | | | | | | |
| 30 s | 5.1 | (4.9 - 5.2) | 5 | (4.8 - 5.1) | 4.7 | (4.5 - 4.8) | 4.9 | (4.7 - 5) |
| 60 s | 7.3 | (7 - 7.5) | 7 | (6.8 - 7.2) | 6.6 | (6.3 - 6.8) | 6.8 | (6.6 - 7.1) |
| 180 s* | 14.2 | (13.7 - 14.7) ^c,d^ | 13.2 | (12.7 - 13.6) | 13 | (12.6 - 13.5) ^a^ | 12.8 | (12.3 - 13.3) ^a^ |
| 300 s* | 19.4 | (18.7 - 20.1) ^b,c,d^ | 17.6 | (17 - 18.3) ^a^ | 17.7 | (17.1 - 18.4) ^a^ | 17.2 | (16.5 - 17.9) ^a^ |
|  | Deaccelerations (distance covered [m]) | | | | | | | |
| 30 s | 24.5 | (23.5 - 25.4) | 23.7 | (22.8 - 24.6) | 23 | (22 - 24) | 22 | (21.2 - 22.9) |
| 60 s | 34.6 | (32.9 - 36.3) | 33.6 | (31.9 - 35.3) | 31.6 | (30 - 33.2) | 30.8 | (29.2 - 32.4) |
| 180 s | 57.6 | (55.1 - 60.1) | 54.2 | (51.9 - 56.6) | 52.1 | (49.6 - 54.6) | 50.4 | (48.1 - 52.8) |
| 300 s* | 77.5 | (74.0 - 81.1) ^c,d^ | 71.7 | (68.5 - 74.9) | 68.7 | (65.5 - 71.9) ^a^ | 66.3 | (63.2 - 69.4) ^a^ |
|  | Deaccelerations (number) | | | | | | | |
| 30 s | 4.8 | (4.7 - 5) | 4.7 | (4.5 - 4.8) | 4.5 | (4.3 - 4.7) | 4.5 | (4.4 - 4.7) |
| 60 s | 6.7 | (6.4 - 6.9) | 6.5 | (6.2 - 6.7) | 6.2 | (5.9 - 6.4) | 6.3 | (6 - 6.5) |
| 180 s* | 12.6 | (12.1 - 13.1) ^c,d^ | 11.9 | (11.5 - 12.4) | 11.4 | (11 - 11.9) ^a^ | 11.3 | (10.9 - 11.8) ^a^ |
| 300 s* | 17.2 | (16.5 - 17.9) ^c,d^ | 15.9 | (15.3 - 16.5) | 15.6 | (14.9 - 16.2) ^a^ | 15.2 | (14.5 - 15.8) ^a^ |
|  | Relative distance covered (m) | | | | | | | |
| 30 s | 73 | (71.1 - 74.8) | 71.8 | (70.5 - 73.2) | 70.7 | (69.1 - 72.3) | 71.2 | (69.8 - 72.7) |
| 60 s | 122.8 | (120.2 - 125.4) | 121 | (119 - 122.9) | 118.4 | (116.0 - 120.9) | 119.2 | (116.8 - 121.6) |
| 180 s | 288.8 | (282.6 - 295) | 280.1 | (274.7 - 285.5) | 285.5 | (279.4 - 291.5) | 276 | (269.7 - 282.3) |
| 300 s | 440.9 | (427.8 - 454.1) | 424.3 | (413.2 - 435.5) | 435.1 | (422.2 - 448) | 414.1 | (402 - 426.1) |
|  | Relative distance covered at the speed zone 1 (stationary / walking) (m)* | | | | | | | |
| 30 s | 64.5 | (62.8 - 66.3) | 61.8 | (60.4 - 63.3) | 61.4 | (59.7 - 63) | 60.7 | (59 - 62.4) |
| 60 s | 98.5 | (95.9 - 101.2) | 94.9 | (92.5 - 97.3) | 92.6 | (89.9 - 95.4) | 93.1 | (90.2 - 96.1) |
| 180 s | 201.9 | (195.9 - 208.0) | 190.9 | (185.4 - 196.5) | 195 | (189 - 201) | 186.9 | (180.2 - 193.5) |
| 300 s* | 291 | (281.4 - 300.7) ^b,d^ | 267.7 | (260.1 - 275.4) ^a^ | 273.4 | (264.6 - 282.3) | 261.7 | (252.2 - 271.1) ^a^ |
|  | Relative distance covered at the speed zone 2 (jogging) (m)* | | | | | | | |
| 30 s | 39.8 | (38.1 - 41.4) | 38.7 | (37.3 - 40.2) | 37.2 | (35.8 - 38.7) | 36.8 | (35.2 - 38.5) |
| 60 s | 49.6 | (47.3 - 51.9) | 49.9 | (47.7 - 52) | 48.6 | (46.5 - 50.7) | 46.4 | (43.9 - 48.8) |
| 180 s | 94.6 | (90.5 - 98.6) | 88.8 | (84.8 - 92.7) | 88.9 | (85.1 - 92.7) | 83 | (78.5 - 87.5) |
| 300 s | 129.6 | (123.7 - 135.5) | 119.2 | (114.1 - 124.3) | 118.7 | (113.6 - 123.7) | 111.1 | (104.9 - 117.3) |
|  | Relative distance covered at the speed zone 3 (running) (m) | | | | | | | |
| 30 s | 14.4 | (13.6 - 15.2) | 13.6 | (12.8 - 14.5) | 13.3 | (12.5 - 14.1) | 12.8 | (12 - 13.6) |
| 60 s | 16 | (15 - 17) | 15.2 | (14.2 - 16.2) | 14.4 | (13.5 - 15.3) | 14 | (13 - 14.9) |
| 180 s | 22.1 | (20.5 - 23.6) | 20.4 | (18.9 - 21.9) | 19.5 | (18 - 21) | 18.2 | (16.7 - 19.7) |
| 300 s | 27 | (25 - 29) | 24.2 | (22.2 - 26.1) | 23 | (21.1 - 24.8) | 21.8 | (19.9 - 23.7) |
|  | Relative distance covered at the speed zone 4 (high-intensity running) (m) | | | | | | | |
| 30 s | 8.5 | (7.9 - 9.1) | 8.5 | (7.8 - 9.3) | 8.2 | (7.4 - 9) | 7.4 | (6.7 - 8.1) |
| 60 s | 8.8 | (8.1 - 9.5) | 8.8 | (8 - 9.5) | 8.4 | (7.6 - 9.2) | 7.6 | (6.8 - 8.4) |
| 180 s | 10 | (9.1 - 10.9) | 9.8 | (8.8 - 10.7) | 9.4 | (8.4 - 10.5) | 8.5 | (7.5 - 9.4) |
| 300 s | 11.3 | (10.1 - 12.4) | 10.8 | (9.7 - 12) | 9.9 | (8.7 - 11.2) | 8.8 | (7.7 - 9.8) |
|  | Relative distance covered at the speed zone 5 (sprinting) (m) | | | | | | | |
| 30 s | 5.4 | (4.6 - 6.1) | 6.2 | (5.1 - 7.2) | 5.6 | (4.4 - 6.8) | 5.5 | (4.6 - 6.4) |
| 60 s | 5.5 | (4.6 - 6.3) | 6.2 | (5.1 - 7.2) | 5.6 | (4.4 - 6.8) | 5.8 | (4.6 - 7.1) |
| 180 s | 5.9 | (5 - 6.8) | 6.7 | (5.3 - 8) | 5.9 | (4.5 - 7.2) | 5.9 | (4.6 - 7.2) |
| 300 s | 6 | (5 - 6.9) | 7 | (5.5 - 8.5) | 5.9 | (4.4 - 7.4) | 5.8 | (4.6 - 7.1) |
| *: The Bayesian ANOVA reported that there was at least a strong evidence (Bayes factor [BF_10_] > 10) to support the alternative hypothesis (H_1_) and a percental error < 0.001.  Post hoc analysis: super-indices indicate that there was at least a strong evidence to support the presence of differences (BF_10_ > 10) against ^a^: quarter 1, ^b^: quarter 2, ^c^: quarter 3 and ^d^: quarter 4. In bold are emphasized those sub-indices whose magnitude of the differences observed were at least moderate (δ > 0.6).  m: meters; s: seconds; km: kilometer; h: hour. | | | | | | | | |
