# Supplementary material for: The Most Demanding Scenarios of Play in Basketball Competition From Elite Under-18 Teams
Source: Front Psychol. 2020 Apr 21;11:552. doi: 10.3389/fpsyg.2020.00552 (PMC7187750; doi:10.3389/fpsyg.2020.00552)
Supplement: Supplementary file 2 [file Data_Sheet_2.docx]

| **Appendix 2: The most demanding scenario of a basketball match play for three different match scores (mean and 95% credible intervals)** | | | | | | | |  |
| --- | --- | --- | --- | --- | --- | --- | --- | --- |
| **Time epoch** | **Match score** | | | | | | |  |
|  | **Winning** | | **Losing** | | | **Drawing** | | |
| Accelerations (distance covered [m]) | | | | | | | |  |
| 30 s | 26.7 | (25.7 - 27.7) | 26.4 | (25.9 - 27) | 25.5 | | (23.1 - 27.8) |  |
| 60 s | 36.4 | (35 - 37.8) | 35.9 | (35 - 36.7) | 35.4 | | (31.6 - 39.2) |  |
| 180 s | 68.1 | (65.4 - 70.8) | 64.8 | (63.2 - 66.4) | 68.7 | | (60.1 - 77.3) |  |
| 300 s | 91.5 | (87.7 - 95.4) | 87 | (84.8 - 89.1) | 78 | | (67.5 - 88.4) |  |
| Accelerations (number) | | | | | | | |  |
| 30 s | 5 | (4.8 - 5.2) | 4.9 | (4.8 - 5) | 5 | | (4.5 - 5.4) |  |
| 60 s | 7 | (6.7 - 7.3) | 6.9 | (6.8 - 7.1) | 6.8 | | (6.1 - 7.5) |  |
| 180 s | 14.1 | (13.5 - 14.6) | 13.1 | (12.8 - 13.4) | 13.2 | | (11.9 - 14.6) |  |
| 300 s | 18.9 | (18.2 - 19.6) | 17.8 | (17.4 - 18.2) | 17.7 | | (15.8 - 19.6) |  |
| Deaccelerations (distance covered [m]) | | | | | | | |  |
| 30 s | 23.2 | (22.3 - 24.1) | 23.5 | (23 - 24.1) | 20.9 | | (18.5 - 23.3) |  |
| 60 s | 33.6 | (24.3 - 31.8) | 32.7 | (31.7 - 33.6) | 28 | | (31.6 - 35.6) |  |
| 180 s | 55.9 | (53.4 - 58.3) | 53.1 | (51.7 - 54.5) | 52.8 | | (44.1 - 61.5) |  |
| 300 s | 74.2 | (70.8 - 77.7) | 70.8 | (68.8 - 72.7) | 60 | | (50.2 - 69.8) |  |
| Deaccelerations (number) | | | | | | | |  |
| 30 s | 4.7 | (4.5 - 4.9) | 4.6 | (4.5 - 4.7) | 4.5 | | (4 - 4.9) |  |
| 60 s | 6.5 | (6.2 - 6.8) | 6.4 | (6.2 - 6.5) | 6.3 | | (5.5 - 7.1) |  |
| 180 s | 12.4 | (11.9 - 12.9) | 11.7 | (11.4 - 12) | 11.4 | | (10.1 - 12.8) |  |
| 300 s | 16.6 | (15.9 - 17.2) | 15.8 | (15.4 - 16.2) | 15.3 | | (13.4 - 17.2) |  |
| Relative distance covered (m) | | | | | | | |  |
| 30 s | 72.1 | (70.9 - 73.2) | 72.3 | (71.2 - 73.3) | 65.9 | | (61.9 - 69.9) ^a^ |  |
| 60 s | 121.1 | (119.4 - 122.9) | 120.5 | (118.7 - 122.2) | 115.5 | | (111.4 - 119.5) |  |
| 180 s | 287.8 | (283.7 - 291.9) | 279.9 | (275.6 - 284.2) | 268.7 | | (253.5 - 283.9) |  |
| 300 s | 434 | (426.7 - 441.4) | 425.9 | (416.8 - 435.1) | 413.1 | | (374.3 - 451.9) |  |
| Relative distance covered at the speed zone 1 (stationary / walking) (m) | | | | | | | |  |
| 30 s | 62.2 | (60.9 - 63.4) | 62.5 | (61.3 - 63.7) | 59.4 | | (56.8 - 61.9) |  |
| 60 s | 95.3 | (93.2 - 97.3) | 95.2 | (93.3 - 97.2) | 90.2 | | (85.8 - 94.6) |  |
| 180 s | 198.2 | (193.8 - 202.6) | 191.6 | (187.2 - 196) | 181 | | (168.8 - 193.1) |  |
| 300 s | 280.3 | (274 - 286.5) | 271.5 | (264.8 - 278.3) | 247.3 | | (228.9 - 265.7) |  |
| Relative distance covered at the speed zone 2 (jogging) (m) | | | | | | | |  |
| 30 s | 38.4 | (37.3 - 39.5) | 38.3 | (37.1 - 39.5) | 36.1 | | (33.5 - 38.7) |  |
| 60 s | 48.8 | (47 - 50.5) | 49 | (47.4 - 50.7) | 45.7 | | (42.3 - 49) |  |
| 180 s | 91.9 | (88.9 - 94.8) | 88.1 | (85.1 - 91.2) | 76.2 | | (69.6 - 82.8) |  |
| 300 s | 123.4 | (119.5 - 127.4) | 119.4 | (115.1 - 123.8) | 100.7 | | (91.5 - 109.8) |  |
| Relative distance covered at the speed zone 3 (running) (m) | | | | | | | |  |
| 30 s | 13.9 | (13.3 - 14.5) | 13.5 | (12.9 - 14.1) | 12.1 | | (10.8 - 13.5) |  |
| 60 s | 15.4 | (14.7 - 16.1) | 14.8 | (14.1 - 15.6) | 13.1 | | (11.6 - 14.7) |  |
| 180 s | 21.2 | (20.1 - 22.4) ^c^ | 19.6 | (18.5 - 20.7) | 16.7 | | (14.5 - 18.9) |  |
| 300 s | 25.6 | (24.2 - 27.1) ^c^ | 23.5 | (22 - 25) | 18.4 | | (15.6 - 21.2) |  |
| Relative distance covered at the speed zone 4 (high-intensity running) (m) | | | | | | | |  |
| 30 s | 8.1 | (7.6 - 8.6) | 8.4 | (7.9 - 9) | 7.2 | | (5.9 - 8.6) |  |
| 60 s | 8.4 | (7.8 - 9) | 8.6 | (8 - 9.1) | 7.4 | | (6 - 8.8) |  |
| 180 s | 9.5 | (8.7 - 10.2) | 9.6 | (8.8 - 10.3) | 8.9 | | (7 - 10.8) |  |
| 300 s | 10.5 | (9.6 - 11.3) | 10.3 | (9.5 - 11.1) | 8.5 | | (6.5 - 10.5) |  |
| Relative distance covered at the speed zone 5 (sprinting) (m) | | | | | | | |  |
| 30 s | 5.7 | (5 - 6.5) | 5.6 | (4.9 - 6.3) | 5.8 | | (3.4 - 8.1) |  |
| 60 s | 5.9 | (5.1 - 6.7) | 5.6 | (4.9 - 6.3) | 5.8 | | (3.4 - 8.1) |  |
| 180 s | 6.3 | (5.4 - 7.2) | 5.9 | (5 - 6.7) | 6.1 | | (3.3 - 8.9) |  |
| 300 s | 6.6 | (5.6 - 7.6) | 5.9 | (5 - 6.7) | 6.3 | | (3.5 - 9.1) |  |
| m: meters; s: seconds; km: kilometer; h: hour | | | | | | | |  |
